# The preparation of several 1,2,3,4,5-functionalized cyclopentane derivatives

**DOI:** 10.3762/bjoc.9.195

**Published:** 2013-08-19

**Authors:** André S Kelch, Peter G Jones, Ina Dix, Henning Hopf

**Affiliations:** 1 Institute of Organic Chemistry, Technical University of Braunschweig, Hagenring 30, D-38106 Braunschweig, Germany, Fax +49-(0)531-391-5288; 2 Institute of Inorganic and Analytical Chemistry, Technical University of Braunschweig, Hagenring 30, D-38106 Braunschweig, Germany; 3 Novartis Pharma AG, CH-4056 Basel, Switzerland

**Keywords:** bromination, cyclopentanes, esterification, permethylcyclopentadiene, polyfunctional compounds, radialenes

## Abstract

With the goal of eventually synthesizing [5]radialene (**3**), the still missing member of the parent radialene hydrocarbons, we have prepared the pentaacetates **21** and **31**, the pentabromide **29** and the hexabromide **32**. In principle these should be convertible by elimination reactions to the desired target molecule.

## Introduction

**Scheme 1:**
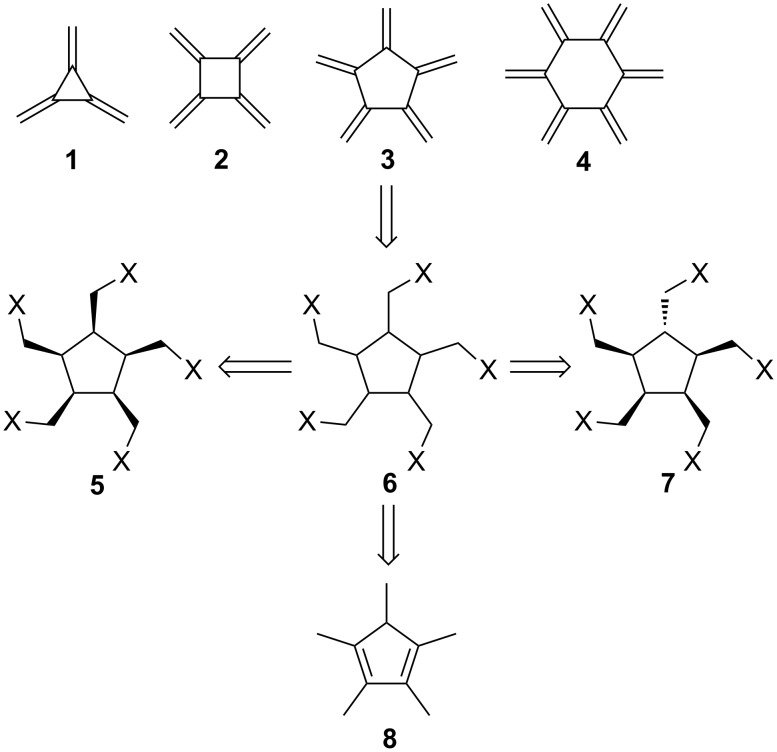
The first members of the [*n*]radialene series and retrosynthesis for [5]radialene (**3**).

Radialenes are cyclic cross-conjugated hydrocarbons that consist solely of semicyclic double bonds. Beginning with [3]radialene (**1**), prepared first by Dorko in 1965 [1], this class of basic oligoenes has been the subject of constantly growing attention by preparative and structural chemists, and – more recently – by material scientists [2–5]. Although derivatives bearing alkyl and aryl and also functionalized substituents, beginning with **1** and ending with [6]radialene (**4**, Scheme 1) are known for the whole series [2–5], among the parent hydrocarbons one representative is still missing: [5]radialene (**3**).

One reason for this absence is certainly the expected low stability of **3**: all its lower and higher analogs are highly reactive hydrocarbons that under normal laboratory conditions polymerize easily and/or readily undergo addition reactions with other compounds – oxygen [2–3], dienophiles [2–3], carbenes [6], and other trapping agents.

Another reason is probably the practical consideration that there are more and higher-yielding preparative routes to three-, four- and six-membered rings than to five-membered ones.

Looking at possible precursors for **3**, it seems reasonable to start with a substrate that contains at least a complete five-membered carbocycle or – even better – a starting material that already displays all ten carbon atoms required in the product. For the first protocol, derivatives such as **6** are possible candidates, not least for the simple reason that in all other reported syntheses of the radialene hydrocarbons [2–3] final steps are involved that employ β-elimination processes – be it of halides, aminoxide derivatives (Cope elimination), acetates (ester pyrolysis), etc. [2–3].

When starting with derivatives of type **6** it should be noted that these compounds can exist in the form of different diastereomers; for example – as shown in **5** – all five substituents could point in the same direction (*cis*,*cis*,*cis*,*cis*-configuration). Alternatively, one (or more) of these functionalized groups could be directed in opposite directions (e.g. *cis*,*cis*,*cis*,*trans*-configuration, as in **7**). Whether this stereochemical difference will have an influence on the ultimate outcome of the multiple elimination process is an open question; but it is likely to influence the rate of this multi-step transformation, which will involve increasingly reactive (unstable) intermediates.

As far as substrates are concerned that possess the complete set of necessary carbon atoms, the commercially available pentamethylcyclo-1,3-pentadiene (**8**) offers itself as the best starting material.

In the present publication we describe the synthesis of the two diastereomeric derivatives **5** and **7** bearing various functional groups (see below), and describe the reaction of **8** with various bromination reagents. Whenever possible we confirm the structures of intermediate compounds or byproducts by X-ray structural analysis.

## Results and Discussion

### *cis*,*cis*,*cis*,*cis*-1,2,3,4,5-Pentakis(hydroxymethyl)cyclopentane (**16**) and derivatives

**Scheme 2:**
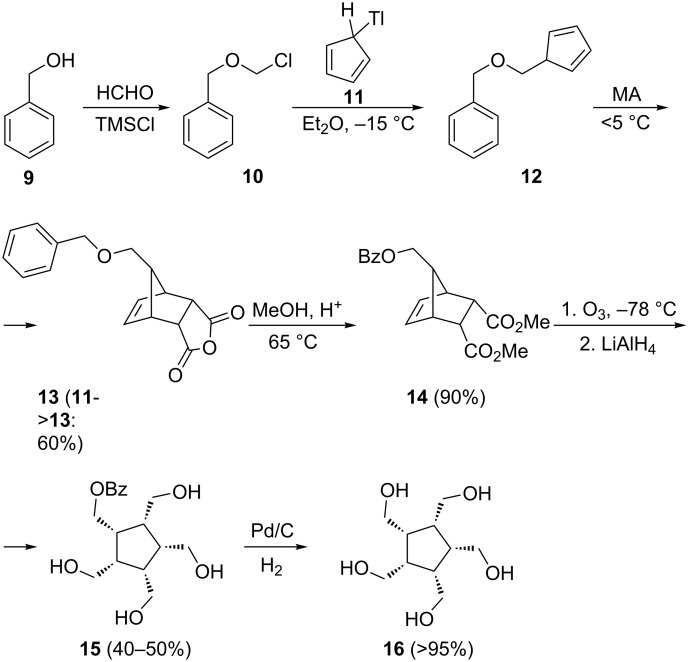
Preparation of *cis*,*cis*,*cis*,*cis*-1,2,3,4,5-pentakis(hydroxymethyl)cyclopentane (**16**) according to Tolbert [7].

The pentaalcohol **16** is a known compound, having first been described by Tolbert and co-workers in 1985 in a short communication [7]. Although in a later contribution these authors reported on the all-*cis* geometry (X-ray analysis) of a derivative of this compound (the pentamesylate, see below) [8]. The experimental and spectroscopic details of the original synthesis were never fully published [9]. This missing information will be presented in this publication (see Supporting Information File 1). The Tolbert synthesis of **16** is summarized in Scheme 2.

The crucial intermediate 5-[(benzyloxy)methyl]cyclopentadiene (**12**) was first prepared from benzyl alcohol (**9**) via the chloro ether **10** and cyclopentadienylthallium (**11**) according to a route developed by Corey and co-workers in their prostaglandin work [10]. Cycloaddition of maleic anhydride (MA) to **12** subsequently afforded the *endo*-adduct **13**, which after esterification provided the diester **14** in excellent yield [7]. This bicyclic adduct was converted by ozonolysis followed by LAH reduction into the all-*cis-*derivative **15**, whose benzyl ether protection group was finally removed by catalytic hydrogenation over palladium on carbon [7–8]. The full experimental details and the complete set of the spectroscopic data of **16** are collected in Supporting Information File 1.

**Scheme 3:**
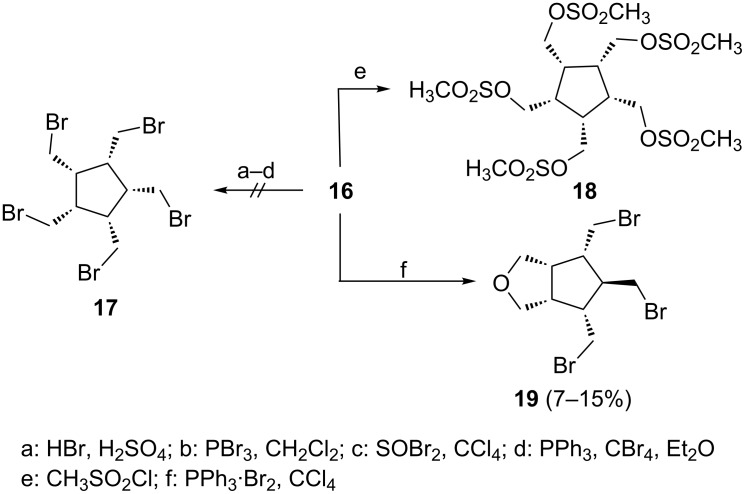
The preparation of derivatives of **16** better suited for nucleophilic substitution and elimination.

The first successful attempt to convert the pentaalcohol into a derivative bearing better leaving groups is Tolbert´s preparation of the pentamesylate **18** (Scheme 3) [8].

**Figure 1:**
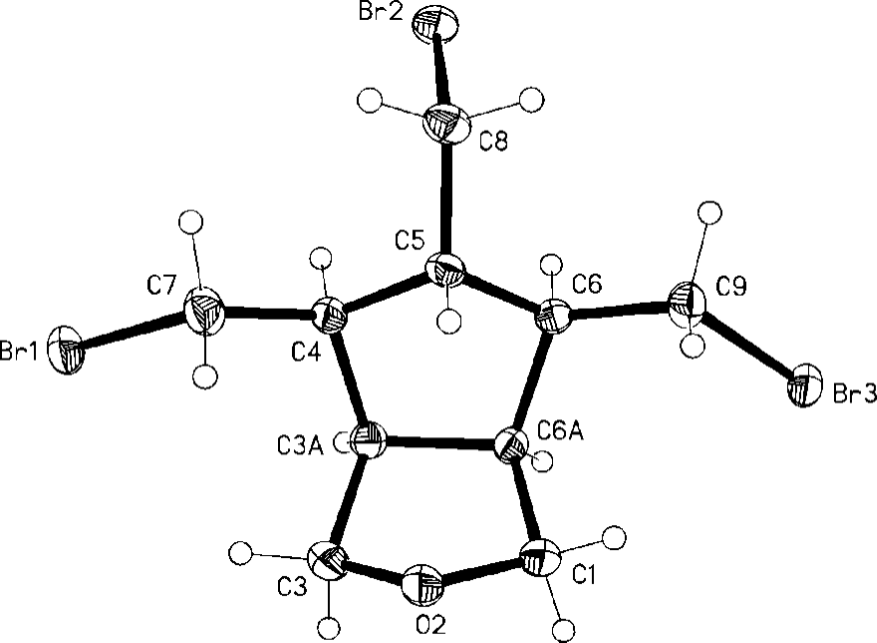
Structure of **19** in the crystal; ellipsoids represent 50% probability levels.

All our attempts to prepare the all-*cis*-pentabromide **17** from **16** failed so far, as is also shown in Scheme 3. Only in one case was a halogenated derivative obtained in poor yield: treatment of **16** with triphenylphosphine–bromine in carbon tetrachloride provided the tribromo ether derivative **19** as a minor component. Surprisingly, this had the *cis*,*cis*,*cis*,*trans*-configuration of the substituents at the five-membered ring, as was shown by single crystal X-ray analysis (Figure 1).

It is unclear at the present time whether the correspondingly configurated pentaalcohol was already present in the starting material **16** as a side-product, which had been overlooked in previous studies, or whether epimerization took place during the attempted conversion of the pentaalcohol to the pentabromide **17**. The complete, structure-confirming spectroscopic and analytical data of **19** can be found in Supporting Information File 1.

In closing this section on the preparation of bromine derivatives of **16**, it should also be mentioned that attempts to prepare the corresponding pentachloride (by treatment with either SOCl_2_ or PCl_3_) and pentaiodide (red P/iodine) also failed. In all these experiments complex product mixtures resulted, the GC–MS analysis of which demonstrated that only partially halogenated derivatives of **16** had been produced; their chromatographic separation failed.

**Scheme 4:**
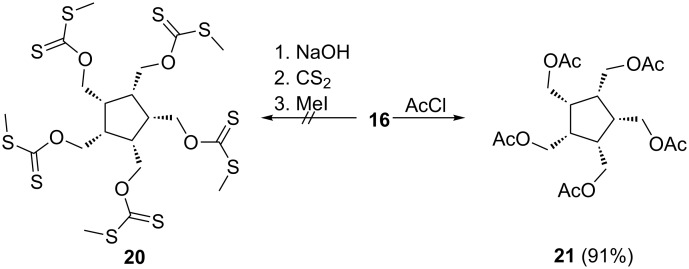
Preparation of the pentaacetate **21** from **16**.

In order to avoid working in solution and subjecting the presumably reactive **5** to extended work-up and purification conditions, we next tried to prepare the xanthate **20** and the acetate **21** from **16**, derivatives that could be subjected to flash vacuum pyrolysis (Scheme 4).

Whereas all our attempts to synthesize **20** failed (see Supporting Information File 1), the pentaester **21** was obtained under standard conditions from **16** by treatment with excess acetyl chloride in excellent yield; its spectroscopic data can be found in the Supporting Information File 1.

### *cis*,*cis*,*cis*,*trans*-1,2,3,4,5- (**26**), *cis,cis,trans,cis*-1,2,3,4,5-Pentakis(methoxycarbonyl)cyclopentane (**27**) and several of their derivatives

For the other two routes to precursors of [5]radialene (**3**) discussed here, the ten carbon atoms of the desired final product were available already at a very early stage of the synthesis.

**Scheme 5:**
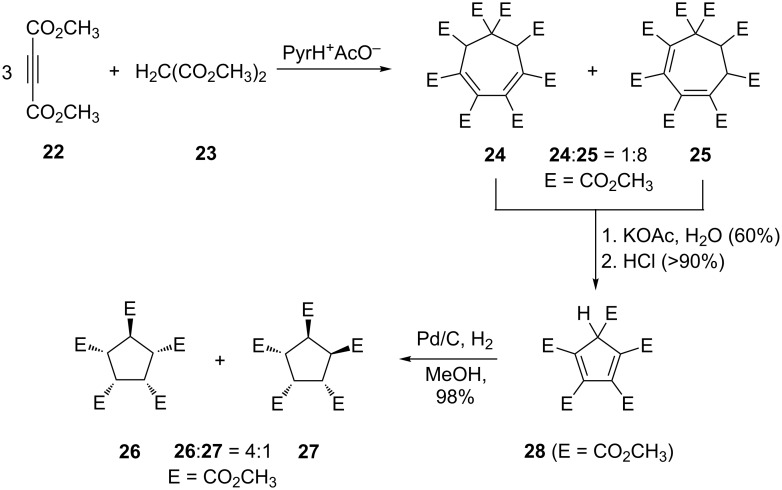
Preparation of the cycloheptadiene octaesters **24**/**25** according to Diels [11] and Le Goff [13], respectively, followed by ring contraction to the cyclopentadiene pentaester **28**.

The first route dates back to studies by Diels, Alder and co-workers [11–12], carried out in the early 1930s and 40s, which were taken up later by Le Goff and his students [13]. These pioneers demonstrated that, on treatment of dimethyl acetylenedicarboxylate (**22**) with dimethyl malonate (**23**) under the catalytic influence of pyridinium acetate, the fully substituted cycloheptadienes **24** and **25** (Scheme 5) were produced; both authors also proposed reaction mechanisms for this complex transformation that can be considered as reasonable even today; for the details the reader is referred to the original literature [12–13].

**Figure 2:**
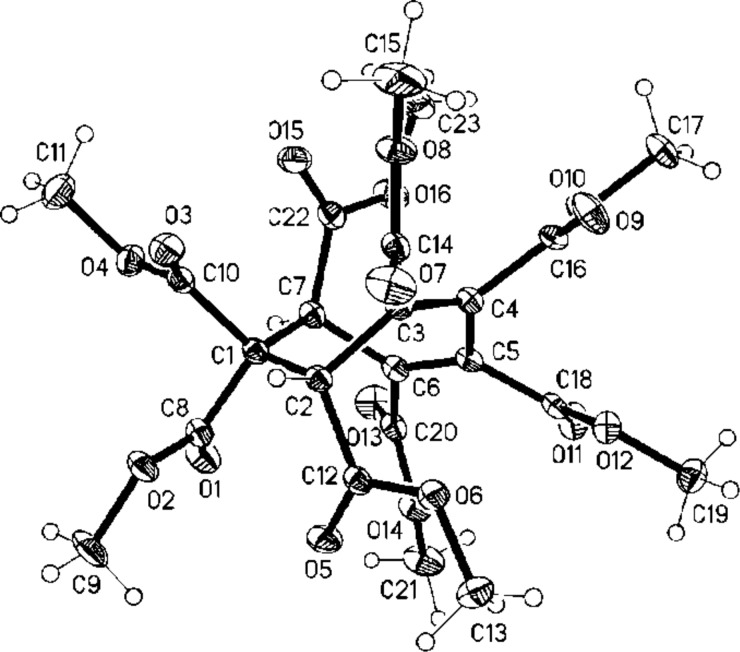
Structure of **24** in the crystal; ellipsoids represent 30% probability levels.

The reproduction of this protocol posed no problems (see Supporting Information File 1). The only difference we noted between our results and those reported in the chemical literature concerns the ratio of **24** to **25**. Whereas Diels reported a ratio of 3:1, and in Le Goff´s hands it was as much as 15:1, we observed the formation of **24** as a minor product (**24**:**25** = 1:8). One reason for this difference could be a pericyclic 1,5-hydrogen shift between the two valence isomers generating different ratios under different preparation and work-up conditions. Another equilibration mechanism could involve a base-induced 1,5-hydrogen shift. Since the mechanism of formation of **24** and **25** was not the main concern of our investigations, we did not clarify these points. The differentiation between the symmetrical **24** and the non-symmetrical **25** by ^1^H NMR spectroscopy is easy and was achieved by Le Goff. More complete and up-to-date spectroscopic data of the two compounds are summarized in Supporting Information File 1. Still, the early workers [11–13] did not distinguish between a possible *meso*-structure (*cis*-orientation of allylic hydrogen atoms) and the chiral (*C*_2_-symmetrical) configuration (*trans*-orientation of hydrogen atoms) of **24**. Fortunately, recrystallization of a **24**/**25**-mixture from diethyl ether provided single crystals of **24** suitable for an X-ray structure investigation which proved the chiral configuration (Figure 2).

The ring contraction of a mixture of **24**/**25**, following the procedure given by Le Goff [13], proceeded as expected and provided the potassium salt of **28** in acceptable yields (60%). From this, free 1,2,3,4,5-pentakis(methoxycarbonyl)cyclopenta-1,3-diene **28** was liberated by hydrochloric acid treatment in excellent yield (>90%, Scheme 5).

**Figure 3:**
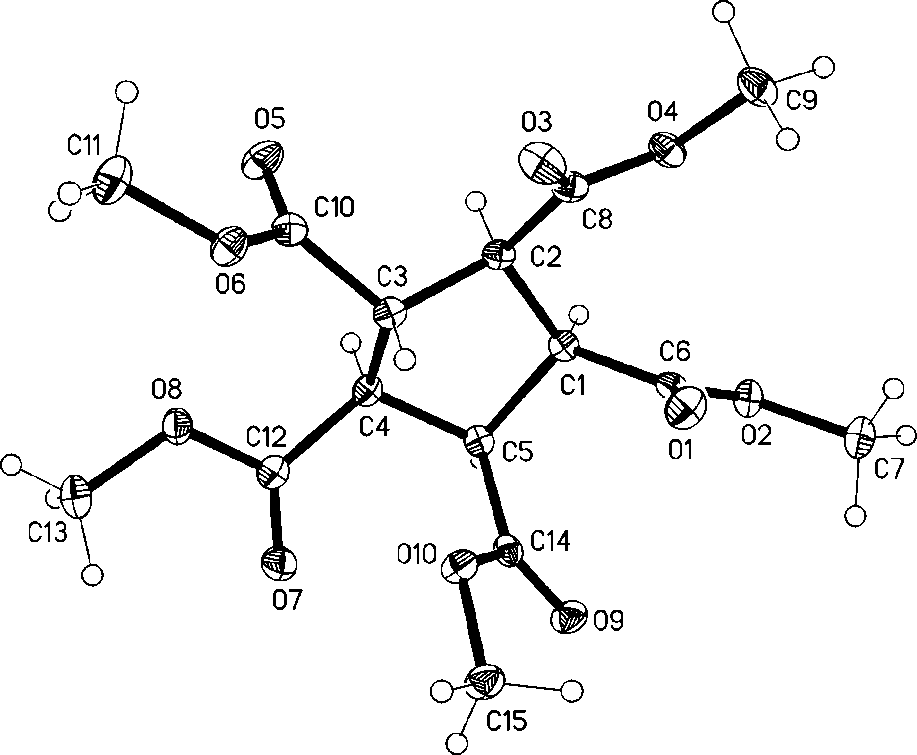
Structure of **26** in the crystal; ellipsoids represent 30% probability levels.

Finally, the hydrogenation of **28** over Pd/C in methanol furnished a mixture of the saturated pentaesters **26** and **27** (ratio 4:1, capillary GC–MS-analysis) in practically quantitative yield (98%). Although we were unable to separate the two components quantitatively by column chromatography on silica gel, the main isomer began to crystallize partially from the highly viscous hydrogenation mixture after standing for several months. The crystals obtained were suitable for X-ray structural analysis, which showed that this isomer has the *cis*,*cis*,*cis*,*trans-*geometry, i.e. **26** (Figure 3).

The structural assignment of the minor hydrogenation product as **27**, which could only be obtained in enriched form (ratio **26**:**27** ca. 1:1) is based on the following considerations. Altogether there exist three hydrogenation products of **28**: the two shown (**26**, **27**) and also the all-*cis*-configurated pentaester, presumably the most highly strained of the three isomers. For symmetry reasons this compound should display two sharp signals in its ^1^H NMR spectrum: one for the tertiary hydrogen atom at the ring and one for the methyl group of its five identical ester groups (ratio 1:3). It should not be difficult to identify these signals in the proton spectrum of a mixture of hydrogenation products because of their relative intensities. Although a large number of signals can indeed be recognized in the corresponding chemical shift regions, none displays an extraordinary intensity. It is hence unlikely that the all-*cis*-pentaester is produced in significant amounts on hydrogenation.

**Scheme 6:**
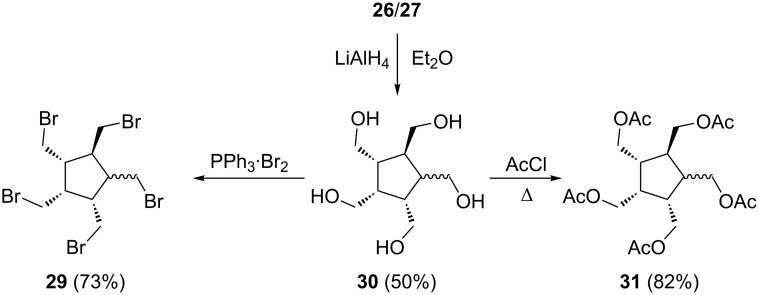
Derivatives derived from the pentaester mixture **26**/**27**.

As far as derivatives of **26** and **27** are concerned, we always worked with the mixture of the two diastereomers (Scheme 6).

Reduction of **26**/**27** with lithium aluminum hydride in either diethyl ether or THF provided the diastereomeric pentaalcohols **30**. The yield of the process is probably higher than the finally isolated 50%. Both isomers are readily soluble in water, and to obtain the pure mixture the crude reduction product was first extracted with diethyl ether or THF. This leaves the inorganic salts and the pentaalcohols as a residual solid. When this is extracted with refluxing methanol or (preferably) isopropanol the desired reduction product **30** slowly dissolves, leaving the salts behind. The removal of the solvent from **30** is difficult and requires extended pumping under high vacuum (2 weeks). Even then the residue shows traces of the originally added solvent (by NMR analysis).

The exact structure assignment of **30** is hampered by the fact that no pure isomer was available. We assume, however, that the isomeric composition is not changed by the LAH reduction (i.e. 4:1 as in the case of the esters **26**/**27**). The signals for the *cis*,*cis*,*cis*,*trans*-pentaalcohol are clearly discernible. The ^1^H NMR spectrum displays three signal groups from δ = 1.63–2.52 in 2:2:1 ratio for the ring protons. In the ^13^C NMR spectrum the ring methine carbon atoms absorb at 45.5, 46.0, and 46.7 ppm, whereas the primary carbon atoms are registered at δ = 60.0, 63.2, and 65.5 ppm. The signals of the minor isomer(s) can hardly be differentiated from the noise.

The bromination of **30** proceeded with varying results; most of the methods that were unsuccessfully used above in the case of **16** also fail for its isomer (HBr, PBr_3_, SOBr_2_ in different halogenated solvents). However, with the triphenylphosphine–bromine complex, a mixture of the pentabromides **29** was obtained in very good yield (73%). The superior results in comparison with **16** probably can be rationalized by a decreased steric shielding of the hydroxymethyl groups in **30**, but are probably also associated with the different solubility of the pentaalcohol mixture. Likewise, the pentaacetate **31** can be obtained from **30** in excellent yield (Scheme 5). As in the case of **16**, no xanthate (see above) could be prepared from **30**.

### Bromination of 1,2,3,4,5-pentamethylcyclopenta-1,3-diene (**8**)

**Scheme 7:**
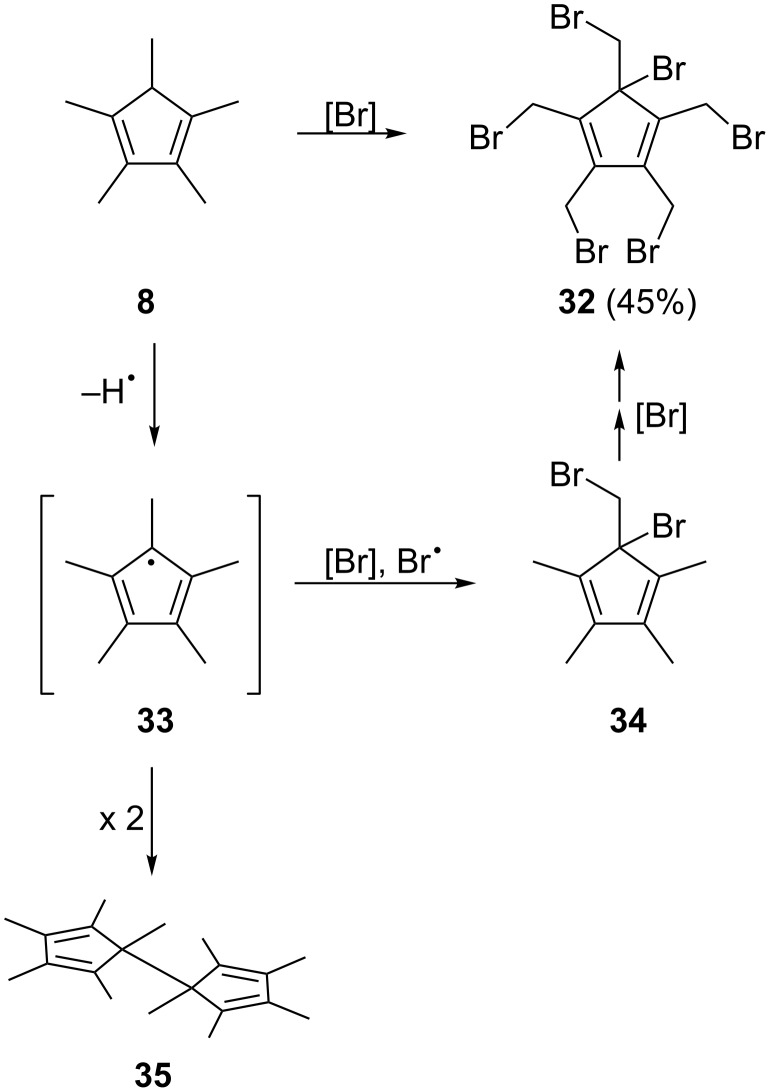
Bromination of 1,2,3,4,5-pentamethylcyclopenta-1,3-diene (**8**).

The other substrate that has all the carbon atoms of the final [5]radialene (**3**) already in place is 1,2,3,4,5-pentamethylcyclopenta-1,3-diene (**8**). Formally this hydrocarbon merely has to be dehydrogenated three times to provide the target molecule. To accomplish this goal we first had to replace these hydrogen atoms by a better leaving group, e.g. bromine as in Scheme 7.

**Figure 4:**
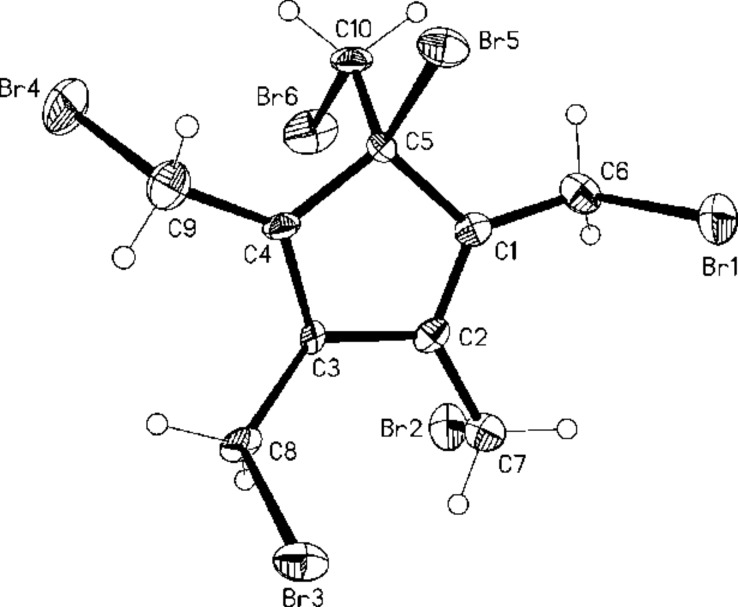
Structure of **32** in the crystal; ellipsoids represent 50% probability levels.

After trying numerous different protocols (Br_2_ addition, NBS in different concentrations and in different solvents, 1,3-dibromo-5,5-dimethylhydantoin) [14] we finally found that excess NBS (6 equivalents) and refluxing in tetrachloromethane at 80 °C gave the best results. Under these conditions the hexabromide **32** was produced in 45% yield. The structure of this cyclopentadiene derivative was derived from its spectroscopic data (see Supporting Information File 1) and also by an X-ray structural analysis (Figure 4).

A rationalization of the route from **8** to **32** is also summarized in Scheme 7. Since in all our bromination experiments we isolated small amounts of a hydrocarbon that, according to mass spectral analysis, has the formula C_20_H_30_, i.e. is a dimer of the starting material, we assume that the permethylcyclopentadienyl radical **33** plays an important role in the bromination reaction. This could not only lead to the bromo derivative **34** in which the final ring hydrogen atom has been substituted, but also to a mixture of hexabromides among which the hexabromide **32** is the major reaction product.

With several derivatives (**18** [7–8], **21**, **29**, **31**, and **32**) that could lead to [5]radialene (**3**) in hand, a synthesis of this long missing hydrocarbon is now within sight.

## Supporting Information

File 1 Experimental procedures and characterization data.
