# Supplementary material for: The preparation of several 1,2,3,4,5-functionalized cyclopentane derivatives
Source: Beilstein J Org Chem. 2013 Aug 19;9:1705–12. doi: 10.3762/bjoc.9.195 (PMC3778403; doi:10.3762/bjoc.9.195)
Supplement: File 1 — Experimental procedures and characterization data. [file Beilstein_J_Org_Chem-09-1705-s001.pdf]

# Supporting Information

# for

# The preparation of several 1,2,3,4,5-functionalized cyclopentane derivatives

André S. Kelch[1], Peter G. Jones[2], Ina Dix[1,3] and Henning Hopf*[1]

Address: [1]Institute of Organic Chemistry, Technical University of Braunschweig, Hagenring 30, D-38106 Braunschweig, Germany, Fax +49-(0)531-391-5288, [2]Institute of Inorganic and Analytical Chemistry, Technical University of Braunschweig, Hagenring 30, D-38106 Braunschweig, Germany and [3]Current address: Novartis Pharma AG, CH-4056 Basel, Switzerland

E-mail: Henning Hopf - h.hopf@tu-bs.de

* Corresponding author

## Experimental procedures and characterization data

**General: Chromatography:** DSC: Polygram SIL G/UV254 (Macherey-Nagel); column chromatography: Kieselgel 60 (70–230 mesh, Merck). Melting points: MEL-TEMP II (Laboratory Devices), uncorr. *NMR*: Bruker AC 200 F ($^{1}$H: 200.1 Hz, $^{13}$C: 50.3 MHz), Bruker AM 400 ($^{1}$H: 400.1 MHz, $^{13}$C: 100.6 MHz, spin multiplicities by the DEPT technique), int. TMS. MS: Finnigan MAT 8430, EI (70 eV). IR: KBr or neat (film): Nicolet 320 FTIR. UV–vis: Hewlett-Packard 8452 Diode Array. Analytical GC: Dani 86.10 HAT Chromtograph with OV-1 cap. Column (20 m). GC–MS: Carlo-Erba HRGC 5160 Chromatograph with Finnigan MAT 4515 (EI, 40 eV). X-ray crystallography: Siemens R3 or Stoe STADI-4 diffractometer. Benzylchloromethyl ether (**10**) was prepared according to [1].

1) *cis*,*cis*,*cis*,*cis*-1,2,3,4,5-Pentakis(hydroxymethyl)cyclopentane (**16**) [2,3]: Cyclopentadienyl thallium (**11**): To a solution of potassium hydroxide (4 g) in water (80 mL) was added thallium sulfate (10 g, 0.02 mol). Under cooling (ice bath) and vigorous stirring was slowly added freshly prepared 1,3-cyclopentadiene (10 mL, 0.13 mol), and the mixture was stirred for a further 1 h. The product was removed by filtration and washed with cold methanol. Drying of the residue under high vacuum (**11** begins to sublime under these conditions!) provided a yellow solid (6.5 g, 65%), m.p. 260–270 °C.

2) 5-Benzyloxymethyl-1,3-cyclopentadiene (**12**) [2]: According to [2,4] cyclopentadienyl thallium (**11**) (22 g, 0.08 mol) was dissolved in anhydrous ether (50 mL) under nitrogen. At −15 °C a solution of **10** (16 g, 0.1 mol) in anhydrous ether (30 mL) was added with vigorous stirring. After 6 h at this temperature, the reaction mixture was filtered through a glass frit into a round-bottomed flask kept at −30 °C. The resulting clear solution can be used without further purification in the subsequent Diels–Alder addition.

3) 10-Benzyloxymethyl-4-oxa-tricyclo[5.2.1.$0^{2,6}$]dec-8-en-3,5-dione (**13**) [2]: To an ice-cold solution of **12** (20 g, 0.108 mol) in diethyl ether (150 mL) was slowly added a solution of maleic anhydride (MA, 10 g, 0.1 mol) in diethyl ether (50 mL). After stirring for 8 h at 0 °C the solution was concentrated under vacuum until the product began to crystallize. The colorless crystals (18.46 g, 65%) were removed by filtration, m.p. 104 °C. $^{1}$H NMR (400.1 MHz, $CDCl_3$): δ = 2.33 (t, $J_{10\text{-H}/11\text{-H}}$ = 7.0 Hz, 1 H, 10-H), 3.33 (d, $J_{11\text{-H}/10\text{-H}}$ = 7 Hz, 2 H, 11-H), 3.43 (m, 2 H, 1-H and 7-H), 3.57 (m, 2 H, 2-H and 6-H), 4.41 (s, 2 H, 13-H), 6.18 (ps-t, 2 H, 8-H and 9-H), 7.26-7.36 ppm (m, 5 H, aromatic-H). $^{13}$C NMR (100.6 MHz, $CDCl_3$): δ = 47.0 (d, C-2 and C-6), 47.7 (d, C-1 and C-7), 64.2 (d, C-10), 66.6 (t, C-11), 73.2 (t, C-13), 127.5 (d, C-Aryl), 127.7 (d, C-17), 128.4 (d, C-Aryl), 132.9 (d, C-8 and C-9), 137.9 (s, C-14), 170.8 ppm (s, C-3 and

C-5). IR (KBr): $\tilde{\upsilon}$ = 3085 (w), 3021 (w), 2997 (w), 2985 (w), 2943 (w), 2933 (w), 2895 (w), 2859 (w), 1857 (m), 1772 (s, C=O), 1454 (m), 1353 (m), 1334 (m), 1293 (m), 1272 (m), 1255 (m), 1240 (m), 1232 (m), 1208 (m), 1137 (m), 1115 (m), 1089 (s), 1008 (m), 942 (m), 910 (s), 767 (m), 702 (m), 628 $cm^{-1}$ (m). UV (acetonitrile): $\lambda_{max}$ (lg ε) = 200 (4.0), 214 nm (3.8). MS (EI, 70 eV): *m*/*z* (%) = 284 (57) [$M^+$], 91 (100).

4*)* Dimethyl benzyloxymethyl-bicyclo[2.2.1]hept-5-en-2,3-dicarboxylate (**14**) [2]: The anhydride **13** (10 g, 35 mmol) was suspended in methanol (100 mL) and 3 to 4 drops of conc. sulfuric acid were added; **13** began to dissolve slowly. The reaction mixture was heated to reflux for 6 h, cooled down, and the solvent was removed in vacuo. The resulting oil was purified/separated by silica gel column chromatography with pentane:diethyl ether = 9:2 as eluent. After standing at room temperature for extended periods of time (months) colorless needles crystallized: 10.36 g (90%), m.p. 65 °C. $^1$H NMR (400.1 MHz, $CDCl_3$): δ = 2.12 (t, *J* = 7.0 Hz, 1 H, 4-H), 3.13 (ps-s, 2 H, 2-H), 3.33 (ps-s, 2 H, 3-H), 3.38 (d, *J* = 7.0 Hz, 2 H, 5-H), 3.60 (s, 6 H, $OCH_3$), 4.42 (s, 2 H, 6-H), 6.14 (m, 2 H, 1-H), 7.22 ppm (m, 5 H, 7-H, 8-H, 9-H, 10-H). $^{13}$C NMR (100.6 MHz, $CDCl_3$): δ = 48.1 (d, C-2), 48.5 (d, C-3), 51.5 (q, $OCH_3$), 59.8 (d, C-4), 67.4 (t, C-5), 73.0 (t, C-6), 127.4 (d, C-8 or C-9), 127.5 (d, C-10), 128.3 (d, C-8 or C-9), 132.2 (d, C-1), 138.2 (s, C-7), 172.5 ppm (s, C=O). IR (KBr): $\tilde{\upsilon}$ = 3064 (w), 3029 (w), 2989 (w), 2950 (m), 2919 (w), 2855 (w), 1746 (s, C=O), 1454 (m), 1436 (m), 1351 (m), 1345 (m), 1338 (m), 1250 (m), 1197 (s), 1168 (m), 1137 (m), 1096 (m), 1077 (m), 1046 (m), 920 (w), 739 (m), 699 $cm^{-1}$ (m). UV (methanol): $\lambda_{max}$ (lg ε) = 258 nm (2.4). MS (EI, 70 eV): *m*/*z* (%) = 330 (5) [$M^+$], 299 (3) [$M^+$ - $OCH_3$], 239 (8), 224 (16), 113 (26), 91 (100). When the esterification was carried out at room temperature, besides the diester **14** a monoester was isolated also (product ratio 2:1 according to $^1$H NMR analysis): $^1$H NMR (400.1 MHz, $CDCl_3$): δ = 2.11 (t, $J_{7\text{-H}/8\text{-H}}$ = 7.0 Hz, 1 H, 7-H), 3.13 (br. ps-s, 2 H, 1-H and 4-H), 3.33 (m, 2 H, 2-H and 3-H), 3.38

(d, $J_{8\text{-H}/7\text{-H}}$ = 7.0 Hz, 2 H, 8-H), 3.57 (s, 3 H, 16-H), 4.42 (s, 2 H, 9-H), 6.07–6.20 (m, 2 H, 5-H and 6-H), 7.23–7.34 ppm (m, 5 H, aryl-H). $^{13}$C NMR (100.6 MHz, $CDCl_3$): δ = 48.0 (d, C-1 or C-4), 48.4 (d, C-1 or C-4), 48.5 (d, C-2 or C-3), 48.7 (d, C-2 or C-3), 51.5 (q, C-16), 59.9 (d, C-7), 67.4 (t, C-8), 73.1 (t, C-9), 127.4 (d, aryl-C), 127.5 (d, C-13), 128.3 (d, aryl-C), 131.7 (d, C-5 or C-6), 133.0 (d, C-5 or C-6), 138.2 (s, C-10), 172.5 (s, C-15), 176.7 ppm (s, C-14). MS (EI, 70 eV): *m*/*z* (%) = 316 (8) [$M^+$], 315 (5) [$M^+$ - H], 284 (3), 239 (2), 224 (6), 210 (11), 113 (9), 105 (11), 91 (100).

5) Ozonolysis of **14** [2]: A solution of **14** (10.0 g, 30 mmol) in dichloromethane (150 mL) was cooled to dry ice temperature. Ozone was passed through the reaction mixture for 20 h at this temperature, and the end of the reaction was recognized by a color change to blue. The solvent was removed in vacuo below 20 °C, and diethyl ether (or THF) was added to the reaction mixture. The resulting solution could be used without further purification for the subsequent reduction.

6) Reduction of the ozonide to the tetraalcohol **15** [5]: The solution from the previous experiment was slowly (2–3 h) added under nitrogen to a suspension of $LiAlH_4$ (3.8 g, 0.1 mol) in diethyl ether (or THF) contained in a round-bottomed flask (250 mL), equipped with reflux condenser and dropping funnel. The reduction took place vigorously so the speed of addition had to be controlled carefully. After the addition was complete the reaction mixture was heated under reflux for 2 h. The mixture was cooled to room temperature and aqueous methanol (50%) was added to destroy excess $LiAlH_4$. The resulting yellow suspension was filtered. The insoluble product was adsorbed by the inorganic salts and to recover as much of it as possible, the filter cake was thoroughly extracted several times with boiling isopropanol (reflux time 1 h each). The resulting extracts were combined, filtered, and the solvent was removed by rotary evaporation: colorless, highly viscous oil, 4.50 g (48%). $^1$H NMR (200.1 MHz, $D_2O$): δ = 1.71 (m, 2 H, H-2/H-5 or H-3/H-4), 2.07 (m, 2 H, H-2/H-5 or H-

3/H-4), 2.35–2.40 (m, 1 H, H-1), 3.51–3.56 (m, 4 H, H-7/H-10 or H-8/H-9), 3.55–3.64 (m, 4 H, H-7/H-10 or H-8/H-9), 3.65–3.71 (m, 2 H, H-6), 4.51–4.54 (m, 2 H, H-11), 7.40 ppm (m, 5 H, H-13, H-14, H-15). $^{13}$C NMR (50.3 MHz, $D_2O$): δ = 45.5 (d), 45.9 (d), 46.9 (d), 59.8 (t, C-6), 62.2 (t, C-7/C-10 or C-8/C-9), 64.6 (t, C-7/C-10 or C-8/C-9), 73.6 (t, C-11), 128.2 (d, C-15), 129.2 (d, C-13 or C-14), 129.5 (d, C-13 or C-14), 138.5 ppm (s, C-12).

7) *cis*,*cis*,*cis*,*cis*-1,2,3,4,5-Pentakis(hydroxymethyl)cyclopentane (**16**): A solution of the tetraol **15** (3.5 g, 11.3 mmol) in methanol (150 mL) was hydrogenated over Pd/C (300 mg) for 4 d at rt. The catalyst was removed by filtration and the solvent was removed in vacuo providing a colorless, highly viscous oil (2.4 g, 95%) which began to crystallize on standing at rt for several weeks; m.p. 50 °C. $^{1}$H NMR (200.1 MHz, $D_2O$): δ = 2.50 (s, 5 H, -CH-), 3.68 ppm (s, 10 H, $CH_2OH$). $^{13}$C NMR (50.3 MHz, $D_2O$): δ = 45.2 (d, -CH-), 60.5 ppm (t, $CH_2OH$). IR (KBr): $\tilde{\upsilon}$ = 3334 (vs, OH), 2974 (m), 2946 (m), 2931 (m), 2921 (m), 2884 (m), 1479 (w), 1452 (w), 1384 (w), 1027 (s), 1013 (s), 704 cm$^{-1}$ (w). UV (methanol): $\lambda_{max}$ (lg ε) = 242 (1.39), 254 nm (1.33). Elemental analysis ($C_{10}H_{20}O_5$, 220.25): calcd. C 54.53 H 9.15; found C 54.49 H 9.63.

8) Bromination of **16** with $PBr_3$ in dichloromethane: To **16** (1.0 g, 4.5 mmol) was added with stirring anhydrous dichloromethane (40 mL), followed by $PBr_3$ (2.2 mL, 6.22 g, 23 mmol) in dichloromethane (20 mL) at rt. The reaction mixture was heated at reflux for 2 d, and hydrolyzed after cooling to rt. The phases were separated, neutralized (sodium bicarbonate solution), and dried (sodium sulfate). The solvent was removed by rotary evaporation, furnishing a yellow oil that turned black within a few days on standing at rt. According to GC–MS analysis the product mixture contained at least 6 main products, none of which had the correct mass for the desired product; no starting material could be recovered. - A very similar result was observed when the solvent in this bromination experiment was changed to DMF.

9) Bromination of **16** with HBr: The pentaol **16** (1.0 g, 4.5 mmol) was dissolved in conc. HBr (20 mL) and conc. sulfuric acid (5 mL) was added to this solution. The mixture was heated to reflux for 24 h under nitrogen, and then cooled to rt. After careful extraction with dichloromethane and neutralization with bicarbonate solution, the combined organic phases were dried (sodium sulfate) and the solvent was removed in vacuo. The slightly brown oil thus obtained soon began to turn black. GC–MS-analysis of the raw product mixture showed that at least 6 products had been formed, none having the correct mass for a pentabromide. $^{1}$H NMR spectroscopy also gave no hint of formation of the desired product (**17**); no substrate could be recovered. – Very similar results were obtained when the bromination was attempted with thionylbromide. – A final bromination experiment of **16** with tetrabromomethane/triphenylphosphine provided a product mixture consisting of three reaction products after work-up (see above), but according to GC–MS analysis the desired **17** was again missing; substrate **16** was also not recovered under these conditions.

10) Preparation of tribromide **19**: Bromination of **16** with $PPh_3 \cdot Br_2$ in $CCl_4$; To a solution of triphenylphosphine (9.8 g, 37 mmol) in anhydrous carbon tetrachloride (100 mL) was slowly added bromine (7.04 g, 2.3 mL, 44 mmol) at 0 °C under nitrogen and the resulting solution stirred for 3 h at this temperature. Subsequently **16** (1.2 g, 6.8 mmol) was added in portions and the reaction mixture heated under reflux for 2 d. After cooling to rt, excess bromination agent was decomposed by the addition of methanol. The precipitate formed was removed by filtration, the solvent was removed from the mother liquor in vacuo, and the remainder was dissolved in trichloromethane. To remove the produced triphenylphosphine oxide, the solution was passed through a Florisil column. The raw product mixture obtained after solvent removal (1.78 g) contained at least three different substances, one of which could be

separated by thin-layer chromatography (silica gel, hexane/ethyl acetate = 9:1, $R_f$ 0.40). The colorless, highly viscous oil slowly began to crystallize, furnishing single crystals suitable for X-ray structural analysis: 200 mg of **29** (7%), m.p. 74 °C. $^1$H NMR (400.1 MHz, $CDCl_3$): δ = 2.69 (m, 3 H, 4-H, 5-H, 6-H), 2.99 (m, 2 H, 3-H, 6-H), 3.48 (m, 4 H, 7-H, 8-H, 9-H), 3.51 (d, *J* = 10.3 Hz, 2 H, 1-H, 3-H), 3.75 (m, 2 H, 7-H, 9-H), 3.92 ppm (d, *J* = 10.3 Hz, 2 H, 1-H, 3-H). $^{13}$C NMR (100.6 MHz, $CDCl_3$): δ = 27.5 (t, C-8), 31.3 (t, C-7, C-9), 45.0 (d, C-5), 47.9 (d, C-4, C-6), 49.2 (d, -CH-), 68.1 ppm (t, -$CH_2$O-). IR (film): $\tilde{\upsilon}$ = 2964 (s), 2934 (s), 2866 (s), 1487 (w), 1440 (s), 1341 (w), 1269 (s), 1222 (s), 1184 (m), 1075 (m), 1057 (s), 906 (m), 814 (m), 732 (s), 646 $cm^{-1}$ (s). UV–vis (acetonitrile): $\lambda_{max}$ (lg ε) = 234 nm (2.68). MS (GC–MS): *m*/*z* (%) = 391 (3) [$M^+$ - H], 313 (22), 311 (44), 309 (22), 283 (8), 281 (16), 279 (8), 201 (31), 199 (24), 149 (15), 131 (27), 119 (100), 105 (67), 91 (76), 79 (55), 67 (42), 53 (38), 41 (33). Elemental analysis ($C_{10}H_{15}Br_3O$, 390.94), calcd C 30.72, H 3.87; found C 30.86, H 3.77. Selected spectroscopic data were obtained for two other products, which, however, could not be obtained in pure form: Substance A: (hexane: ethyl acetate = 9:1; $R_F$ 0.75): 200 mg. $^1$H NMR (400.1 MHz, $CDCl_3$): δ = 2.03 (m, -$CH_2$-), 3.43 ppm (m, -$CH_2$-). $^{13}$C NMR (100.3 MHz, $CDCl_3$): δ = 30.9 (t), 32.5 ppm (t). Substance B (hexane:ethyl acetate = 9:1; $R_F$ 0.45). 60 mg. $^1$H NMR (400.1 MHz, $CDCl_3$): δ = 2.27 (m, 1 H, -CH-), 2.33 (s, 2 H, -CH-), 2.82 (m, 2 H, -CH-), 3.59 ("t", 2 H, -$CH_2$-), 3.69 (m, 2 H, -$CH_2$-), 3.75 ("d", 2 H, -$CH_2$-), 3.81 ("d", 4 H, -$CH_2$-). $^{13}$C NMR (100.6 MHz, $CDCl_3$): δ = 30.1 (t, -$CH_2$Br), 30.2 (t, -$CH_2$Br), 41.6 (d, -CH-), 44.2 (d, -CH-), 44.8 (d, -CH-), 61.6 (t, -$CH_2$O-). MS (GC–MS): *m*/*z* (%) = 394 (15) [$M^+$], 392 (36) [$M^+$], 390 (31) [$M^+$], 388 (14) [$M^+$], 313 (16), 311 (32), 309 (18), 283 (4), 281 (9), 279 (6), 201 (33), 199 (28), 187 (24), 185 (26), 149 (17), 131 (16), 119 (100), 105 (69), 91 (65), 79 (42), 67 (32), 53 (31), 41 (22). The NMR- and MS spectra indicate that substance B might be an isomer of **19**.

11) Attempts to prepare the pentaxanthates **20**: According to the method of Whitmore [6]: In a three-necked flask (100 mL) **16** (1.0 g, 4.5 mmol), finally dispersed sodium hydroxide (1.0 g, 25 mmol), diethyl ether (40 mL), and carbon tetrachloride (5 mL) were placed. The mixture was stirred at rt until a finely divided suspension had formed; subsequently a solution of carbon disulfide (1.5 mL, 1.9 g, 25 mmol) in diethyl ether (5 mL) was added within 30 min keeping the internal temperature below 30 °C. After stirring for 3 h at rt, a solution of methyl iodide (1.6 mL, 3.5 g, 25 mmol) in ether (5 mL) was added and the reaction mixture heated to reflux for 24 h. After cooling, the mixture was poured unto ice water (100 mL) and extracted carefully with dichloromethane. The organic phases were combined, washed with water, and dried (sodium sulfate). Removal of the solvent in vacuo furnished a red-brown, smelly oil (raw yield: 50 mg). The expected product **20** could not be detected. Repetition of the experiment using the mixture **30** also failed, as did a route to xanthates proposed by Wynberg [7].

12) 1,2,3,4,5-Pentakis(acetoxymethyl)cyclopentane (**21**): A solution of **16** (1.0 g, 4.5 mmol) in acetyl chloride (30 mL, 33.1 g, 0.42 mol) was heated to reflux for 2 d. Excess acetyl chloride was removed in vacuo as completely as possible, the remaining raw product mixture was decomposed with water (30 mL). Aqueous bicarbonate solution was added until a slightly basic pH was obtained, and the mixture was extracted several times with diethyl ether. The combined organic phases were washed thoroughly with water, and after drying (sodium sulfate), the solvent was removed by rotary evaporation. The resulting slightly yellow oil was purified by silica gel column chromatography with ethyl acetate: 1.76 g (91%) of a colorless oil with a fruity smell. $^1$H NMR (400.1 MHz, $CDCl_3$): δ = 2.06 (s, 15 H, $CH_3O$-), 2.68 (br.-s, 5 H, -CH-), 4.22 ppm (s, 10 H, -$CH_2$OAc). $^{13}$C NMR (100.6 MHz, $CDCl_3$): δ = 20.9 (d, -CH-), 41.5 (q, $CH_3$), 62.4 (t, -$CH_2$-), 170.6 ppm (s, -C(O)-). IR (KBr): $\tilde{\upsilon}$ = 2958 (w,

$CH_2$), 2903 (w), 1741 (s, C=O), 1469 (w), 1450 (w), 1369 (m), 1240 (s), 1036 (m), 976 $cm^{-1}$ (w). UV (acetonitrile): $\lambda_{max}$ (lg ε) = 210 (2.51), 220 (2.42), 230 (2.12), 244 nm (1.47). MS (GC–MS): *m*/*z* (%) = 429 (0.5) [$M^+$ - H], 267 (2), 225 (5), 208 (6), 190 (15), 148 (34), 130 (100), 118 (16), 91 (8), 79 (4). ($C_{20}H_{30}O_{10}$, 430.43): calcd C 55.81, H 7.02; found C 55.25, H 6.79.

13) 1,1,2,3,4,5,6,7-Octakis(methoxycarbonyl)-3,5-cycloheptadiene (**24**) and 1,1,2,3,4-5,6,7-octakis(methoxycarbonyl)-2,4-cycloheptadiene (**25**) [8]: To a solution of dimethyl acetylenedicarboxylate (**22**, 38 g, 0.27 mol) and dimethyl malonate (**23**, 11.7 g, 0.09 mol) in anhydrous diethyl ether (60 mL) was added with vigorous stirring a mixture of pyridine and acetic acid (1.5 g each), keeping the reaction temperature below 45 °C. After heating to reflux, the reaction mixture was cooled to rt, and the resulting solid precipitate removed by filtration and washed with diethyl ether. To the solid was added methanol (150 mL), the mixture brought to boil, and the hot solution filtered. Product **24**, dissolved in the filtrate, crystallized on cooling. The residue was recrystallized from methanol (500 mL) and furnished **25**. Yield of **24**: 4.6 g (9%), m.p. 225 °C. $^1$H NMR (200.1 MHz, $CDCl_3$): δ = 3.69 (s, 6 H, $OCH_{3)}$, 3.70 (s, 6 H, $OCH_{3)}$, 3.74 (s, 6 H, $OCH_3$), 3.89 (s, 6 H, $OCH_3$), 5.08 ppm (br.-s, 2 H, allyl-H). $^{13}$C NMR (50.3 MHz, $CDCl_3$): δ = 52.8 (q, $OCH_3$), 53.1 (q, $OCH_3$), 53.2 (q, $OCH_3$), 53.3 (q, $OCH_3$), 73.8 (s, C-1), 140.4 (s, C=C), 164.4 (s, **C**$O_2CH_3$), 165.4 (s, **C**$O_2CH_3$), 167.8 (s, **C**$O_2CH_3$), 169.2 ppm (s, **C**$O_2CH_3$). UV–vis (methanol): $\lambda_{max}$ (lg ε) = 272 nm (3.66). m.p.: 225 °C. Yield of **25**: 35.5 g (71 %), m.p. 182 °C. $^1$H NMR (200.1 MHz, $CDCl_3$): δ = 3.70 (s, 3 H, $OCH_3$), 3.72 (s, 3 H, $OCH_3$), 3.73 (s, 3 H, $OCH_3$), 3.74 (s, 6 H, $OCH_3$), 3.79 (s, 3 H, $OCH_3$), 3.80 (s, 3 H, $OCH_3$), 3.85 (s, 3H, $OCH_3$), 4.71 (d, $J_{6\text{-H},7\text{-H}}$ = 1.3 Hz, 1 H, 7-H), 5.15 ppm (d, $J_{6\text{-H}, 7\text{-H}}$ = 1.3 Hz, 1 H, 6-H). $^{13}$C NMR (50.3 MHz, $CDCl_3$): δ = 44.3 (d, C-7), 52.7 (q, $OCH_3$), 52.8 (q, $OCH_3$), 52.9 (q, $OCH_3$), 53.0 (q, $OCH_3$), 53.2 (q, $OCH_3$), 53.3 (q, $OCH_3$), 53.8 (q, $OCH_3$), 53.9 (q, $OCH_3$), 63.6 (s, C-1), 63.7

(d, C-6), 132.2 (s, C=C), 134.5 (s, C=C), 142.9 (s, C=C), 144.6 (s, C=C), 163.5 (s, **C**$O_2CH_3$), 164.0 (s, **C**$O_2CH_3$), 166.0 (s, **C**$O_2CH_3$), 166.1 (s, **C**$O_2CH_3$), 167.4 (s, **C**$O_2CH_3$), 168.1 (s, **C**$O_2CH_3$), 169.7 (s, **C**$O_2CH_3$), 170.2 ppm (s, **C**$O_2CH_3$). UV–vis (methanol): $\lambda_{max}$ (lg ε) = 234 (3.18), 242 (3.58), 251 (3.55), 276 nm (3.63). IR (KBr): $\tilde{\upsilon}$ = 3013 (w), 2960 (m, CH-Valenz), 1754 (s, C=O), 1742 (s, C=O), 1734 (s, C=O), 1687 (w), 1607 (w), 1436 (m), 1295 (s), 1266 (s), 1245 (s), 1221 (s), 1130 (m), 1004 (m), 991 $cm^{-1}$ (m). MS (EI, 70 eV): *m*/*z* (%) = 558 (4) [$M^+$], 526 (100), 495 (44), 467 (26), 435 (50), 423 (37), 370 (28), 324 (27), 293 (21), 279 (15), 171 (26), 159 (17), 59 (14).

14) 1,2,3,4,5-Pentakis(methoxycarbonyl)cyclopentadiene (**28**): To a solution of **25** (2 g, 3.6 mmol; alternatively **24** may be used) in water (10 mL) potassium acetate (3.3 g, 34 mmol) was added, and the solution heated to reflux for 2 h. The hot solution was filtered, and the filtrate cooled to room temperature. The crystallized product was filtered off and dried over $P_4O_{10}$. The potassium salt of **28** was obtained as a yellow solid (decomp. p. ca 170 °C) in 70% yield (1.0 g). $^1$H NMR (200.1 MHz, $D_2O$): δ = 3.78 (s, 15 H, $OCH_3$). MS (FAB, NBA, pos.): *m*/*z* (%) = 433 (100) [$M^{++}$ K], 394 (8) [$M^+$], 231 (8), 192 (16), 137 (10), 124 (9). MS (FAB, NBA, neg.): *m*/*z* (%) = 548 (0.4) [$M^{++}$ $H^{++}$ NBA], 547 (1.2) [$M^{++}$ NBA], 546 (1.1) [$M^{++}$ NBA, - H], 355 (100) [$M^+$ - K], 309 (16), 251 (8), 193 (4), 153 (3) [NBA]. The dried potassium salt of **28** was dissolved in water (5 mL) and the solution treated with conc. HCl until all free acid had deposited. The produced cyclopentadiene derivative **28** was dried for 24 h under high vacuum. The slightly yellow solid (m.p. 148 °C) took up a brownish color on standing in air; total yield: 0.5 g (42%). $^1$H NMR (200.1 MHz, $CDCl_3$): δ = 3.80 (ps-s, 3 H, $OCH_3$), 3.90 (ps-s, 6 H, $OCH_3$), 4.00 ppm (ps-s, 6 H, $OCH_3$). $^1$H NMR (200.1 MHz, $D_2O$): δ = 3.75 (s, 15 H, $OCH_3$). $^{13}$C NMR (50.3 MHz, $D_2O$): δ = 53.2 (q, $OCH_3$), 117.5 (s, C-ring), 169.8 ppm (s, **C**$O_2CH_3$). IR (KBr): $\tilde{\upsilon}$ = 3039 (w), 3022 (w),

3014 (w), 3010 (w), 2959 (m), 2929 (w), 2854 (w), 1733 (s, C=O), 1712 (s, C=O), 1608 (s), 1461 (s), 1356 (s), 1235 (s), 1219 (s), 1097 (m), 1003 (m), 989 $cm^{-1}$ (m). UV (methanol): $\lambda_{max}$ (lg ε) = 262 (4.66), 280 (4.07), 296 nm (4.14). MS (EI, 70 eV): *m*/*z* (%) = 356 (8) [$M^+$], 325 (7), 324 (44), 294 (16), 293 (100), 263 (12), 235 (28), 205 (8), 175 (6), 147 (4), 118 (7), 91 (2), 69 (4), 59 (6).

15) *cis,cis,cis,trans*-1,2,3,4,5-Pentakis(methoxycarbonyl)cyclopentane (**26**) and *cis,cis,trans,cis*-1,2,3,4,5-pentakis(methoxycarbonyl)cyclopentane (**27**): To a solution of **28** (3 g, 8.4 mmol) in methanol (120 mL) was added palladium on charcoal (0.5 g) and the suspension was hydrogenated at rt and atmospheric pressure for 4 d. The solvent was removed in vacuo, and the remaining oil passed through a silica gel column. No separation of isomers was observed. However, from the highly viscous product mixture isomer **26** partially crystallizes, providing single crystals (m.p. 55 °C) suitable for X-ray structural analysis. Isomer **27** could only be enriched, not purified fully. Yield (isomeric mixture): 2.81 g (93%). Spectroscopic data of **26**: $^1$H NMR (200.1 MHz, $CDCl_3$): δ = 3.42 (dd, $J_{1\text{-}H/2\text{-}H}$ = 8.6 Hz, 1 H, 2-H), 3.48 (dd, 1 H, 3-H), 3.69 (s, 6 H, 8-H or 9-H), 3.71 (s, 6 H, 8-H or 9-H), 3.77 (s, 3 H, 7-H), 4.12 ppm (t, $J_{1\text{-}H/2\text{-}H}$ = 8.5 Hz, 1 H, 1-H). $^{13}$C NMR (50.3 MHz, $CDCl_3$): δ = 47.3 (d, C-1), 48.1 (d, C-3), 48.8 (d, C-2), 52.1 (q, C-8 or C-9), 52.4 (q, C-8 or C-9), 52.7 (q, C-7), 170.7 (s, C-5 or C-6), 170.8 (s, C-5 or C-6), 174.0 ppm (s, C-4). IR (KBr): $\tilde{\upsilon}$ = 2989 (w), 2961 (m), 2924 (w), 2917 (w), 2853 (w), 1736 (vs, C=O), 1439 (s), 1337 (s), 1283 (s), 1267 (s), 1216 (s), 1120 (m), 1111 (m), 1056 (m), 1039 (m), 1009 (m), 971 (m), 906 (m), 841 (m), 836 $cm^{-1}$ (m). UV (acetonitrile): $\lambda_{max}$ (lg ε) = 210 (2.78), 230 (2.50), 240 (2.22), 260 (1.81), 286 (1.69), 300 (1.65), 342 nm (1.56). - MS (EI, 70 eV): *m*/*z* (%) = 360 (4) [$M^+$], 329 (100), 300 (21), 271 (78), 209 (65), 197 (44), 183 (32), 151 (61), 139 (18), 124 (17), 93 (13), 79 (10), 65 (8) [$M^+$ - 5 x $CO_2CH_3$], 59 (27). Elemental analysis ($C_{15}H_{20}O_{10}$, 360.29), calcd. C 50.00, H 5.59; found C 50.21, H 5.72.

16) *cis,cis,cis,trans*- and *cis,cis,trans,cis*-Pentakis(hydroxymethyl)cyclopentane (**30**): A solution of a mixture of **26** and **27** (2.5 g, 7.0 mmol) in diethyl ether (80 mL) and benzene (60 mL) was reduced with $LiAlH_4$ (1 g, 26.0 mmol) which had been placed into the sleeve of a Soxhlet extraction apparatus. After refluxing for 2 d, the cooled reaction mixture was treated with aqueous methanol (50%, 8 mL). The solid residue was removed by filtration and extracted several times with methanol. The mother liquors were combined and the solvents were removed in vacuo furnishing a slightly yellow, highly viscous oil: 0.31 g (20 %) of the isomer mixture **30**. Spectroscopic data: $^1$H NMR (200.1 MHz, $D_2O$): δ = 1.63–1.73 (m, 1 H, -CH-), 2.05–2.20 (m, 2 H, -CH-), 2.38–2.52 (m, 2 H, -CH-), 3.58–3.82 ppm (m, 10 H, $CH_2OH$). $^{13}$C NMR (50.3 MHz, $D_2O$): δ = 45.5 (d, ring), 46.0 (d, ring), 46.7 (d, ring), 60.0 (t, $CH_2OH$), 63.2 (t, $CH_2OH$), 65.5 (t, $CH_2OH$). IR (KBr): $\tilde{\upsilon}$ = 3334 (s, OH), 2970 (m), 2945 (m), 2921 (m), 2884 (m), 1020 cm$^{-1}$ (m). UV (methanol): $\lambda_{max}$ (lg ε) = 242 nm (1.37). Elemental analysis ($C_{10}H_{20}O_5$, 220.25), calcd C 54.53, H 9.15; found C 54.43, H 7.22. In an alternative reduction experiment $LiAlH_4$ (3.0 g, 78.0 mmol) in diethyl ether (50 mL) was added to a mixture of **26**/**27** (6.0 g, 18.0 mmol) in diethyl ether (100 mL) , and the reaction mixture heated to reflux for 2 d. After work-up (see above), 1.98 g (50%) of a mixture of **30** was obtained, identical in its spectroscopic properties with those of the pentaols described above.

17) 1,2,3,4,5-Pentakis(bromomethyl)cyclopentane **29** (diastereomeric mixture): Bromination of **30** with $PPh_3 \cdot Br_2$ in $CCl_4$: To a solution of triphenylphosphine (13.2 g, 50.0 mmol) in tetrachloromethane (140 mL) was added under nitrogen a solution of bromine (3 mL, 9.3 g, 58 mmol) in tetrachloromethane (30 mL). The suspension was stirred for 3 h at 0 °C and then heated to 70 °C. The pentaol mixture **30** (0.85 g, 3.9 mmol) was added in small portions, and when the addition was complete the reaction mixture was heated to reflux for 2 d. Excess bromination reagent was

destroyed by adding a few mL of methanol, and the solid residue was removed by filtration. The solvent was removed in vacuo and the residue taken up in dichloromethane. The resulting solution was passed through a Florisil column (removal of triphenylphosphine oxide) and the solvent was removed by rotary evaporation. The resulting colorless, highly viscous oil (1.53 g, 73%) turns yellow even in the ice-box in the dark within 12 h. The isomeric products **29** could neither be separated by high-vacuum distillation nor silica gel chromatography. $^{1}$H NMR (400.1 MHz, $CDCl_3$): δ = 2.23 (m, 1 H, -CH-), 2.34 (br. s, 3 H, -CH-), 2.60 (m, 2 H, -CH-), 2.72 (ps-t, 2 H, -CH-), 2.90 (m, 2 H, -CH-), 3.45 (t, *J* = 10.0 Hz, 1 H, $-CH_2Br$), 3.50-3.65 (m, 17 H, $-CH_2Br$), 3.75 ppm (d, *J* = 3.2 Hz, 2 H, $-CH_2Br$). $^{13}$C NMR (100.6 MHz, $CDCl_3$): δ = 28.8 (d, -CH-), 32.3 (d, -CH-), 32.4 (d, -CH-), 35.2 (d, -CH-), 35.7 (d, -CH-), 38.3 (d, -CH-), 46.0 (t, $-CH_2Br$), 46.2 (t, $-CH_2$ Br), 46.6 (t, $-CH_2Br$), 46.9 (t, $-CH_2Br$), 47.3 (t, $-CH_2Br$), 49.1 (t, $-CH_2Br$). MS (GC–MS, peak 1, retention time: 12:55 min): *m*/*z* (%) = 455 (1) [$M^+$ - Br], 377 (14), 375 (50), 373 (51), 371 (15), 295 (48), 293 (100), 291 (51), 213 (37), 211 (33), 133 (21), 119 (17), 105 (15), 91 (19), 79 (20), 53 (32). MS (GC–MS, peak 2, retention time: 14:18 min): *m*/*z* (%) = 455 (1) [$M^+$ - Br], 377 (12), 375 (40), 373 (40), 371 (11), 295 (49), 293 (100), 291 (51), 213 (50), 211 (40), 133 (22), 119 (14), 105 (16), 91 (21), 79 (22), 53 (36).

18) Diastereomeric mixture of pentakis(acetoxymethyl)cyclopentane (**31**): A solution of **30** (diastereomeric mixture, 1.0 g, 4.5 mmol) in acetyl chloride (30 mL, 33.1 g, 0.42 mol) was heated to reflux for 2 d. Work-up as described above provided a mixture of the two esters **31**, which could not be separated by either column or preparative gas chromatography: 1.6 g (82%), colorless oil with a fruity smell. $^{1}$H NMR (400.1 MHz, $CDCl_3$): δ = 1.85–1.92 (m, -CH-), 2.00–2.08 (m, -CH-), 2.06 (s, $-CH_3$), 2.07 (s, $-CH_3$), 2.08 (s, $-CH_3$), 2.22–2.33 (m, -CH-), 2.59–2.63 (m, -CH-), 4.09–4.17 ppm (m, 10 H, $-CH_2O-$). $^{13}$C NMR (100.6 MHz, $CDCl_3$): δ = 20.8 (q, $-CH_3$), 20.9

(q, $-CH_3$), 20.9 (q, $-CH_3$), 41.7 (d, -CH-), 42.0 (d, -CH-), 42.2 (d, -CH-), 43.1 (d,-CH-), 43.3 (d, -CH-), 43.4 (d, -CH-), 61.7 (t, $-CH_2O-$), 63.8 (t, $-CH_2O-$), 64.0 (t, $-CH_2O-$), 65.4 (t, $-CH_2O-$), 65.6 (t, $-CH_2O-$), 66.0 (t, $-CH_2O-$), 170.6 (s, C=O), 170.7 (s, C=O), 170.8 (s, C=O), 170.9 ppm (s, C=O). IR (KBr): $\tilde{\upsilon}$ = 2968 (m), 2940 (m), 2921 (m), 1747 (s, C=O), 1743 (s, C=O), 1739 (s, C=O), 1471 (m), 1436 (m), 1392 (m), 1370 (s), 1236 (s), 1233 (s), 1036 (s), 975 (m), 930 (m), 820 (m), 606 $cm^{-1}$ (m). UV (acetonitrile): $\lambda_{max}$ (lg ε) = 210 (2.50), 220 (2.40), 230 (2.10), 245 nm (1.45). MS (EI, 70 eV): *m*/*z* (%) = 430 (1) [$M^+$], 387 (1), 327 (4), 285 (2), 225 (4), 208 (5), 190 (20), 148 (16), 130 (46), 118 (21), 105 (10), 91 (8), 79 (7), 43 (100). - ($C_{20}H_{30}O_{10}$, 430.43): calcd C 55.81, H 7.02; found C 55.21, H 6.59.

19) Bromination of 1,2,3,4,5-pentamethylcyclopenta-1,3-diene (**8**).

a) Bromination with excess NBS: To a solution of **8** (NBS, 1.0 g, 7.5 mmol) in carbon tetrachloride (50 mL) was added under nitrogen *N*-bromosuccinimide (8.9 g, 50 mmol). After stirring for 1 h at rt, the reaction mixture was heated at 80 °C for 2 h. After stirring at rt overnight, succinimide was removed by filtration and the solvent was evaporated in vacuo. The remaining deep-orange oil was separated by column chromatography (silica gel, tetrachloromethane). The resulting highly viscous yellow oil began to crystallize in the freezer. Recrystallization from pentane/diethyl ether provided single crystals of **32** suitable for X-ray analysis: 2.06 g (45%), m.p. 72 °C. $^1$H NMR (200.1 MHz, $CDCl_3$, int TMS): δ = 4.42 (ps-d, 2 H), 4.39 (ps-d, 2 H), 4.39 (ps-d, 2 H), 2.23 (ps-d, 2 H, all $=C-CH_2Br$), 4.00 ppm (s, 2 H, $C-CH_2Br$). $^{13}$C NMR (50.3 MHz, $CDCl_3$, int TMS): δ = 20.0 (t), 20.6 (t), 31.3 (t), 65.6 (s), 141.7 (s), 141.9 ppm (s). IR (KBr): $\tilde{\upsilon}$ = 3017 (w), 2962 (w), 1445 (m), 621 $cm^{-1}$ (s). UV (acetonitrile): $\lambda_{max}$ (lg ε) = 214 nm (3.82). MS (70 eV): *m*/*z* (%) = 609 (2, $M^+$, isotope pattern: 603 (5), 605 (32), 607 (76), 609 (100), 611 (73), 613 (27), 615 (5), 529 (37), 449 (11), 369 (56), 290 (34), 209 (85), 130 (100).

b) Bromination with excess 1,3-dibromo-5,5-dimethylhydantoin [4]. To a solution of **8** (1.0 g, 7.5 mmol) in tetrachloromethane (50 mL) was added under nitrogen and under stirring 1,3-dibromo-5,5-dimethylhydantoin (7.4 g, 27 mmol). After heating the reaction mixture to reflux for 2 h, it was worked-up as described above yielding 2.3 g (50%) of **32**, m.p. 72 °C. The spectroscopic data were identical with those described above.

20) X-Ray structure determinations: Numerical data are summarised in Table S1. Intensity data were registered at −130 °C using monochromated MoKα radiation (λ = 0.71073 Å) on a Stoe STADI-4 (**24**, **32**) or a Bruker SMART 1000 CCD diffractometer (**19**, **26**). Absorption corrections for **19** and **26** were performed on the basis of multi-scans. Structures were refined anisotropically on $F^2$ using the program SHELXL-97 [9]. Hydrogen atoms were included as rigid methyl groups or using a riding model. *Special features and exceptions*: Methyl hydrogens at C9, C13, C15, C17 and C23 of **24** and C7 of **26** were refined as ideally disordered hexagons of half-occupied hydrogen sites. Hydrogen atoms H01-05 of **26** were refined freely. The rather high *R* values of **32** are probably attributable to residual absorption errors.

Crystallographic data have been deposited with the Cambridge Crystallographic Data Centre as supplementary publications no. CCDC-935101 (**19**), -935102 (**24**), -935103 (**26**), -935104 (**32**). Copies of the data can be obtained free of charge from www.ccdc.cam.ac.uk/data_request/cif.

**Table S1:** Crystallographic data for compounds **19**, **24**, **26**, **32**.

| Compound | **19** | **24** | **26** | **32** |
|---|---|---|---|---|
| Formula | $C_{10}H_{15}Br_3O$ | $C_{23}H_{26}O_{16}$ | $C_{15}H_{20}O_{10}$ | $C_{10}H_{10}Br_6$ |
| $M_r$ | 390.95 | 558.44 | 360.31 | 609.64 |
| Habit | colourless prism | colourless prism | colourless tablet | yellow prism |
| Cryst. size (mm) | 0.35×0.18×0.17 | 0.75×0.55×0.5 | 0.4×0.35×0.2 | 0.45×0.35×0.3 |
| Crystal system | Monoclinic | monoclinic | monoclinic | triclinic |
| Space group | $P2_1/c$ | $P2_1/c$ | $P2_1/c$ | $P\bar{1}$ |
| Cell constants: | | | | |
| *a* (Å) | 8.6899(10) | 9.928(2) | 10.0652(18) | 8.531(3) |
| *b* (Å) | 11.4710(12) | 10.816(2) | 16.4466(12) | 8.672(4) |
| *c* (Å) | 12.6401(15) | 23.694(5) | 11.0200(8) | 11.029(4) |
| α (°) | 90 | 90 | 90 | 88.03(2) |
| β (°) | 100.057(4) | 91.73(3) | 112.257(3) | 71.95(2) |
| γ (°) | 90 | 90 | 90 | 76.88(2) |
| $V$ (Å$^3$) | 1240.6 | 2543.3 | 1685.0 | 754.9 |
| $Z$ | 4 | 4 | 4 | 2 |
| $D_x$ (Mg m$^{-3}$) | 2.093 | 1.458 | 1.420 | 2.682 |
| μ (mm$^{-1}$) | 9.7 | 0.13 | 0.12 | 15.9 |
| $F$(000) | 752 | 1168 | 760 | 560 |
| $T$ (°C) | −130 | −130 | −130 | −130 |
| $2\theta_{max}$ | 56.6 | 50 | 52.6 | 50 |
| Refl. measured | 18361 | 5778 | 10654 | 3381 |
| Refl. indep. | 3075 | 4499 | 3449 | 2654 |
| $R_{int}$ | 0.046 | 0.039 | 0.041 | 0.039 |
| Parameters | 127 | 360 | 251 | 146 |
| $wR(F^2$, all refl.) | 0.070 | 0.149 | 0.092 | 0.161 |
| $R(F, >4\sigma(F))$ | 0.028 | 0.056 | 0.035 | 0.061 |
| $S$ | 0.98 | 1.02 | 1.01 | 1.07 |
| max. Δ/ρ (e Å$^{-3}$) | 1.1 | 0.26 | 0.26 | 1.7 |

## References


1. Shipov, A. G.; Savost´yanova, I. A.; Baukov, Y. I. *Zh. Obshch. Khim.* **1989,** *59,* 1204; J. Gen. Chem. USSR, 1989, 59, 1067.
2. Tolbert, L. M.; Gregory, J. C.; Brock, C. P. *J. Org. Chem.* **1985,** *50,* 548. doi:10.1021/jo00204a030
3. Brock, C. P.; Gregory, J. C.; Tolbert, L. M. *Acta Crystallogr., Sect. C* **1968,** *42,* 1063. doi:10.1107/S0108270186093460
4. Corey, E. J.; Koelliker, U.; Neuffer, J. *J. Am. Chem. Soc.* **1971,** *93,* 1489. doi:10.1021/ja00735a031
5. Gholami, M.; Tykwinski, R. R. *Chem. Rev.* **2006,** *106,* 4997. doi:10.1021/cr0505573
6. Whitmore, F. C.; Simpson, C. T. *J. Am. Chem. Soc.* **1933,** *55,* 3809. doi:10.1021/ja01336a060
7. de Groot, A.; Evenhuis, B.; Wynberg, H. *J. Org. Chem.* **1968,** *33,* 2214. doi:10.1021/jo01270a010
8. Le Goff, E.; LaCount, R. B. *J. Org. Chem.* **1964,** *29,* 423. doi:10.1021/jo01025a043
9. Sheldrick, G. M. *Acta Crystallogr., Sect. A* **2008,** *64,* 112. doi:10.1107/S0108767307043930
